# A Methodology to Measure Glucose Metabolism by Quantitative Analysis of PET Images

**DOI:** 10.1007/s41666-024-00172-7

**Published:** 2024-09-25

**Authors:** Patrizia Vizza, Elena Succurro, Giuseppe Pozzi, Pietro Hiram Guzzi, Giuseppe Lucio Cascini, Giuseppe Tradigo, Pierangelo Veltri

**Affiliations:** 1 Department of Medical and Surgery Sciences, Magna Græcia University, Catanzaro, 88100 Italy; 2 Department of Electronics, Information and Bioengineering, Politecnico di Milano, Milan, 20133 Italy; 3 Department of Clinical and Experimental Medicine, Magna Græcia University, Catanzaro, 88100 Italy; 4 Department of Theoretical and Applied Sciences, University e-Campus, Novedrate, 22060 Italy; 5 DIMES Department, University of Calabria, Rende, 87036 Italy

**Keywords:** PET image, Volume of interest (VOI), Large vessel structure, Quantitative analysis, Glucose metabolic rate (MRGlu)

## Abstract

Positron emission tomography (PET) with F-18 fluorodeoxyglucose (FDG) tracer is the standard clinical technique to measure myocardial and vessel metabolism and viability and to investigate the metabolic syndrome associated with cardiovascular diseases. The quantitative analysis of PET images allows one to study the cardiovascular physiological processes, by extracting quantitative parameters from the analysis of the tracer kinetic. Here, we propose a new methodology to quantify and evaluate the evolution of glucose metabolism inside the myocardium and the large vascular structures over time. We merge and analyze PET and CT cardiac images, extracting different volumes of interest (VOI) and performing quantitative measurements. To validate it, we apply the methodology to merge images of the aorta vessel for patients affected by metabolic syndrome. The application of the proposed approach to the use case reveals a correlation between administered drugs and metabolic syndrome, measuring the glucose metabolic rate (MRGlu) in both the myocardium and aorta. The proposed methodology can be used to evaluate some cardiovascular risk indexes of diabetic patients, too. The proposed methodology can also be deployed to analyze other application domains.

## Introduction

Diabetes mellitus is a chronic disorder of glucose metabolism, and it represents one of the major risk factors for cardiovascular diseases (CVD), related to multifactorial and complex pathophysiological pathways. Despite the widespread investigations, its reasons are only partially understood. The identification and the study of the physio/pathological processes beyond the glucose mechanism are needed to prevent the increased cardiovascular disease risk associated with diabetes. The quantitative analysis of cardiac glucose metabolism represents a useful approach to measure the metabolic alterations in the setting of cardiovascular diseases [1]. Positron emission tomography (PET) images with 18F-fluorodeoxyglucose (18-FDG) provide us with information on the biological processes that underlie the metabolic heart disease [2]. To address the need for accurate quantification of the metabolic rate of glucose (MRGlu), the here proposed methodology leverages the definition and extraction of volumes of interest (VOIs) using merged PET and CT images. The resulting methodology exploits one of the most used bioimage analysis tools (namely, the PMOD platform [3]), enriched using a semi-automatic VOI extraction, thus supporting physicians to quantify and study the MRGlu.

We deploy the methodology to measure MRGlu in the aorta vessel over a cohort of patients from the University Magna Græcia Clinical Hospital. We validate the results in terms of clinical indexes, showing that the methodology supports physicians in comparing the effects of different drug administration on patients affected by diabetes, and measure indexes that evaluate some cardiovascular risk factors.

The novel proposed methodology allows one to (i) merge PET/CT images for the quantification of glucose metabolism and (ii) select volumes of interest (VOIs) for metabolic measurements in the considered district (e.g., myocardium, aorta, or other districts). VOI selection can be performed in a semi-automated way starting from a single user-defined point.

The paper is structured as follows: Section 2 reports some background considerations and the state of the art of available frameworks to study MRGlu; Section 3 reports the proposed methodology; Section 4 reports the deploying of the methodology in real cases, where experimental results validate the proposed methodology; finally, Section 5 sketches out some possible research directions and further applications for the proposed methodology.

## Background and Related Works

In this section, we explore the essential preliminaries of our work and provide an overview of the state-of-the-art approaches that are currently guiding the research efforts in the field.

### Background

Positron emission tomography (PET) is a diagnostic tool useful in clinical practice to perform diagnosis, staging, and therapy response evaluation with an increasingly important role in research and clinical applications [4]. Integration of PET and computer tomography (CT) images by using a PET/CT scanner allows one to collect and analyze both anatomic and metabolic or functional information in vivo, in one single scanning session [5].

Visual inspection of PET images allows the physician to evaluate many clinical conditions, but the recent approaches of PET as a quantitative tool are used to provide an accurate and less observer-dependent measurement for prognosis, diagnosis, and response to treatment [6, 7].

The use of quantitative analysis of PET images allows one to perform some measurements of the physiological processes by using mathematical kinetic modeling techniques [6, 8, 9]. This analysis allows one to study the disease, evaluate and manage the treatment response, and carry out some comparisons between patients. Similarly, bioimages can be used to study cardiovascular functions and to evaluate the myocardial tissue and the coronary flow, by performing a quantitative analysis of heart parameters [10–12]. The quantification of both functional and structural cardiac and myocardium parameters is used in clinical studies. For this reason, PET imaging is used in cardiovascular studies to perform accurate, global and regional measurements of myocardial perfusion and myocardial blood flow [8].

PET quantitative analysis of cardiac images is used to evaluate the myocardial blood flow and the glucose metabolism and to extract diagnostic and prognostic information on coronary artery disease (CAD) [13]. The kinetic modeling represents the standard of most quantification methods for dynamic PET images [14]. For example, the Patlak model is commonly used for studies with tracers undergoing irreversible trapping, e.g., fluorodeoxyglucose (18F-FDG) [15].

Quantitative cardiac PET analysis with Fluorine-18 labeled fluorodeoxyglucose (18F-FDG) tracer is the most accurate non-invasive diagnostic method to characterize CAD. This methodology is also able to evaluate some non-coronary myocardial pathologies and to contribute to the characterization of cardiomyopathies [10]. The 18F-FDG is the most commonly used PET tracer; its uptake is related to metabolic activity, and therefore, it helps to assess the metabolic viability of coronary artery disease. Cardiac 18F-FDG PET studies are performed to determine the standardized uptake value (SUV) and the myocardial metabolic rate of glucose (MRGlu). Dynamic FDG PET quantitatively assesses the regional metabolic rate of glucose (MRGlu) to extract more detailed information about myocardial metabolism aiming to characterize coronary artery behavior [16].

### Related Works

Many works propose quantitative studies based on the analysis of PET images. In cardiac applications, Ko [16] proposes a study to prospectively quantify the MRGlu in the myocardium with different perfusion-metabolism patterns. Zuo et al. in [17] investigate appropriate dynamic scan and kinetic modeling protocols for efficient quantification of myocardial glucose transport. Nakajo et al. in [15] propose a contribution of using a kinetic model to dynamic 18F-FDG PET/CT to evaluate the risk of clinical events in cardiac sarcoidosis.

Henriksenin [18] uses the PET image analysis for quantifying cerebral glucose metabolic rate (CMRglc), also based on the aorta images, by finding a high correlation between CMRglc and the aorta glucose level. Zhang et al. [19] present a framework based on PET images able to segment the left ventricle cavity and myocardium improving the cardiac function assessment. Gullberg et al. in [20] implement a methodology to estimate regional myocardial maps in terms of cardiac efficiency useful to assess and evaluate heart disease.

Lin et al. in [21] investigate the value of 18F-FDG PET/CT imaging by fusing morphological information provided by CT and functional details from CT image fusion aiming to perform the SUV analysis improving a higher sensitivity, specificity, and accuracy of diagnosis in the context of thyroid lesions. Boussion et al. in [22] propose an image fusion framework working with a discrete wavelet-based image merging, to incorporate functional information with structural details performing quantitative analysis directly on the fused images.

The literature reports on several software packages offering kinetic analysis for PET images [9, 23], as well as on related pipelines [24]; nevertheless, none of the available tools is capable of measuring the metabolic glucose rate value over user-defined VOIs in human districts. By using the here proposed methodology, the user is capable of focusing the analysis on VOIs of cardiac districts by using the merged PET and CT images (i.e., structural and functional images). The PMOD commercial tool is the most widely used kinetic modeling tool, offering kinetic analysis functions to facilitate clinical research [3]. We use PMOD as the running environment where the proposed methodology is defined; PMOD is exploited as a plug-in of the workflow, to compute the MRGlu values in user-defined districts (i.e., VOIs). The methodology supports the user to study the district of interest by combining information extracted both from structural (CT) and functional (PET) images.

Almost all the contributions in the literature propose the quantitative cardiac PET analysis by evaluating mainly the myocardium, and they generally work on SUV extraction instead of the MRGlu value. Moreover, to the best of our knowledge, there are few papers addressed to the study of MRGlu value in large vessel structures, e.g., into the aorta [25–28].

## Methods and Methodology

**Fig. 1:**
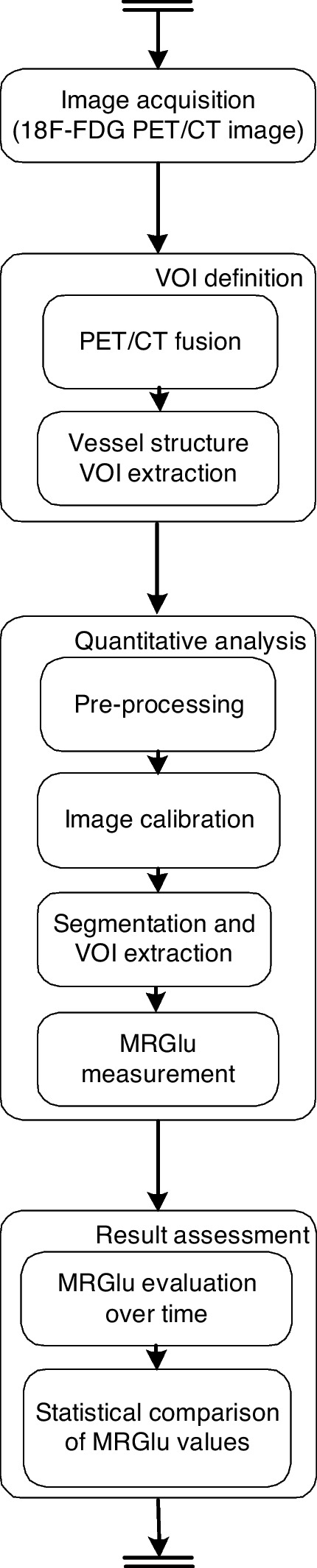
Workflow of the proposed methodology for the quantitative assessment of MRGlu in the large vessel structures: one step (e.g., VOI definition) may include some sub-steps (e.g., PET/CT fusion). Note that VOI definition and quantitative analysis are performed on one single patient; result evaluation is twofold, considering both one single patient and the entire population of patients

We aim to define a new methodology to investigate and quantify the evolution of glucose metabolism inside different anatomical districts (e.g., the myocardium and the large vascular structures). In a nutshell, the methodology consists of data and image acquisition, image processing, and finally statistical and measurement analysis. The workflow of the proposed methodology is reported in Fig. 1.

The methodology is composed of four main steps: (i) image acquisition, (ii) VOI definition for large vessel structures, (iii) quantitative analysis, and (iv) result evaluation.

The *first* step consists of the acquisition of PET and CT images for each patient, useful to analyze the functional features (via the PET) and the structural features (via the CT), simultaneously [29, 30]. PET and CT images allow one to detect the glucose uptake at metabolic and structural levels, respectively. Thus, the PET and CT merged information allows one to perform in the considered districts an accurate characterization of glucose metabolism, which will be performed in the subsequent analysis (i.e., next steps).

The *second* step focuses on the identification of the volumes of interest (VOIs), useful for performing quantitative analysis of the images.

Movement correction has been applied to the PET images, in order to correct the patient’s movement  [31, 32]. The methodology implements a rigid matching algorithm that generates no tissue deformation in the image.

**Fig. 2:**
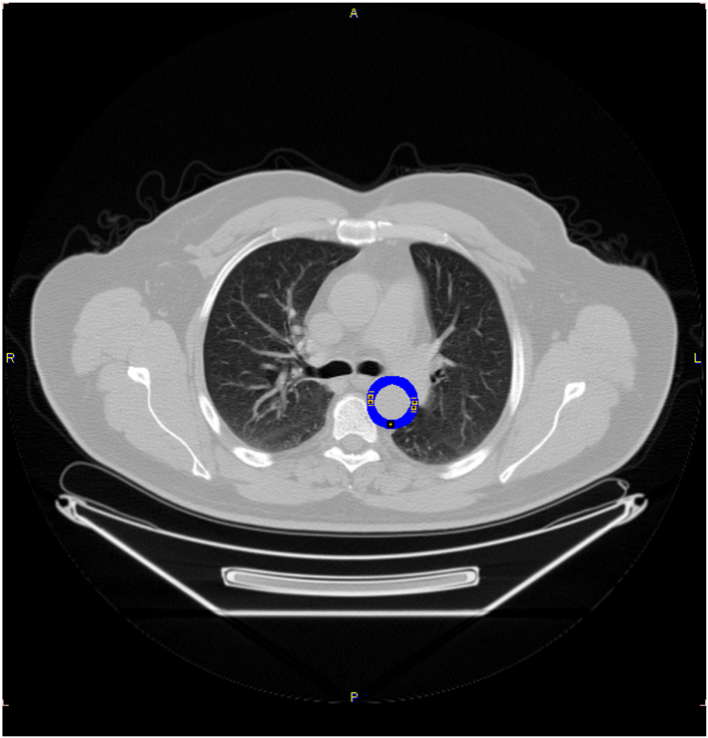
An example of the aorta VOI extraction on the image resulting from the fusion of CT and PET images of a diabetic patient. The blue circle represents the aorta VOI

To select VOIs, the structural and functional slices (PET and CT) are merged. In this step, the vessel structures are selected by following a semi-automated procedure. This step allows the physician to perform a semi-automatic VOI segmentation, supporting the user in the personalized definition of the volume of interest concerning the specific district to be investigated. Indeed, the user may select an anatomical point (e.g., bronchial bifurcation selected for the aorta VOI) to allow automatic VOI selection using the image supporting tool (e.g., PMOD in our case). The methodology then extracts a well-defined vessel structure, which is mapped on the PET image to perform quantitative analysis and calculate the MRGlu values. For instance, the first two steps produce the aorta-extracted VOI section shown in Fig. 2 where a snapshot of the aorta VOI (the blue circle of the figure) is reported.

The *third* step refers to the quantitative analysis, allowing one to calculate the MRGlu values in the analyzed districts. The quantitative analysis is a graphical analysis that uses a linear regression technique to analyze the pharmacokinetics (evolution over time) of a tracer (18F-FDG tracer in our case) in a studied district. Compartmental models can be used to measure MRGlu values varying during the acquisition of sequenced images. The proposed methodology implements the Patlak compartmental model [33], for the quantitative assessment of the regional MRGlu with 18F-FDG. Myocardium and aorta image-derived input functions have been chosen for Patlak analysis to assess the MRGlu uptake values in both districts (i.e., myocardium and aorta VOI), respectively.

For instance, for the cardiac district, the quantitative analysis is performed by using the following sub-steps:

- Pre-processing, to reduce the image noise. In the case of images regarding the myocardium, noise reduction refers to the dynamic heart activity acquisition step.
- Image calibration, to map human district compartments into standard templates (e.g., vessel or heart district alignment w.r.t. district templates).
- Segmentation and district VOI extraction.
- Evaluation of the MRGlu values in the VOIs. The evaluation is performed by analyzing VOIs from images over time by the application of a compartmental model (e.g., the Patlak model).

The *fourth* step refers to the evaluation of the acquired results: the evaluation can be single-patient-oriented, i.e., considering the measurements pre- and post-administration of the drug to one single patient, as well as population-oriented, considering all the measurements on all the patients.

The methodology includes a statistical module to validate the quantitative analysis in terms of MRGlu values. This allows one to validate the correlation of MRGlu values measured in different vessel structures. In the case of drug/multiple drug administration, this step allows one to compare MRGlu values measured during the administration phases. For example, the ANCOVA test is included in the fourth step and allows one to study the follow-up conditions after different drug administrations. For example, for studies regarding the cardiac district, this allows one to evaluate the MRGlu in the myocardium and the aorta vessel, evaluating the effects of two different drugs.

Moreover, a multiple linear regression module is included in the methodology, to relate measured parameters in the case of multiple relevant indexes, as in the case of risk factors related to metabolic diseases. The multiple linear regression is expressed by the multiple correlation coefficient *R* in the $[0 \dots 1]$ range. An *R* value close to 1 indicates a higher predictive power of the independent variable in predicting the dependent variables. For instance, in the case of the MRGlu evaluation in the cardiac district, the multiple linear regression module allows one to estimate the effects of each independent variable (e.g., cardiovascular risk factors) on the dependent variable t (e.g., MRGlu in the aorta vessel) in the studied district.

The above-reported workflow has been integrated and used into the PMOD software platform (Version 3.806). PMOD is a software tool commonly used to perform quantitative analysis of bioimages, and it is widely used in clinical diagnosis as well as in scientific research. The functionalities of PMOD have been integrated with the proposed methodology in vessel structures VOI assessment and MRGlu value extraction. PMOD allows one to analyze the myocardium in an automatic procedure although, to the best of our knowledge, the tool does not support the automatic study of large vessels.

The methodology proposed and implemented in PMOD can manage large vessel structures in terms of VOI definition and metabolism values extraction and compare computed values with the MRGlu of myocardium, in terms of different clinical conditions and cardio-metabolic risk factors. Thus, the methodology for quantitative analysis of MRGlu uses functionalities of PMOD, such as the one that manages the fusion of CT and PET images into one unique coordinate space and that supports VOI extraction. Without loss of generality, the proposed methodology can also be coupled to any other general-purpose tool performing similar functionalities: e.g., some open source tools such as 3DSlicer, caliPER, and OsiriX can be deployed to perform the quantitative analysis of PET images as per the proposed methodology [34–37].

## Validation of the Methodology

We deployed and validated the here proposed methodology in a real-case scenario. We tested the methodology on a cohort of patient data, including 20 diabetic adult subjects with one or more cardio-metabolic risk factors, enrolled by the Internal Medicine Unit of the University of Catanzaro Medical Hospital. Table 1 reports the mean and standard deviation values for the 13 cardiovascular risk factors that have been identified, for all the considered patients [38].

We used the methodology to support physicians in studying a real case, consisting of measuring the effects of the administration of two drugs (namely, empagliflozin and glimepiride) on patients affected by type 2 diabetes. Patients performed PET and CT exams at the Diagnostic and Nuclear Medicine Unit of the Hospital University of Catanzaro. Image acquisition has been performed by using a hybrid General Electric PET/CT scanner (GE Discovery ST8-2D PET scanner). PET images have been reconstructed in a 128$\times$128 matrix, and a correction for decay and attenuation based on co-recorded CT data has been applied.

**Table 1:** Cardiovascular risk factors for patients

Cardiovascular risk factors	Value
Age	56.9 ± 7.49 years
Insulin-stimulated glucose disposal (LeanM)	3.20 ± 1.84 mg/min x Kg FFM
Body mass index (BMI)	31.19 ± 4.55 kg/m$^2$
Waist circumference (W)	107.25 ± 9.95 cm
Systolic blood pressure (sBP)	126.50 ± 11.87 mmHg
Diastolic blood pressure (dBP)	78.35 ± 11.03 mmHg
Pulse pressure (PP)	49.35 ± 9.24 mmHg
Fasting plasma glucose (FPG)	142.15 ± 40.05 mg/dL
Fasting plasma insulin (FPIns)	11.76 ± 6.18 mU/mL
Erythrocyte sedimentation rate (ESR)	13.00 ± 12.36 mm/hour
C-reactive protein (PCR)	3.28 ± 2.75 mg/L
Pulse wave velocity (PWV)	6.90 ± 1.25 m/s
Aortic root diameter (Ao)	3.59 ± 0.41 cm

The recruited patients are affected by type 2 diabetes with one or more cardio-metabolic risk factors. Eligible subjects are recruited according to deep inclusion and exclusion criteria, among which: age between 30 and 70 years, family history of diabetes, dysglycemia, hypertension, dyslipidemia, overweight/obesity, end-stage renal disease, and many others [39]. The bioimages acquisition procedure required that (a) the subjects receive a priming dose of insulin followed by continuous insulin infusion, (b) the blood glucose level is maintained constant by infusing glucose at varying rates, and (c) glucose metabolized is calculated during a clamp examination.

Patients have been divided into two groups, i.e., *Group1* and *Group2*. Two different protocols have been applied: Empagliflozin drug has been administered to *Group1*, and glimepiride has been administered to *Group2*. PET and CT images have been acquired for each patient before and after the treatment (i.e., *pre*, *post*). We then applied the proposed methodology to evaluate respectively the aorta MRGlu value (indicated as *MRGluAorta*) and the myocardium MRGlu value (indicated as *MRGluMyoc*). We recall that the proposed methodology is independent of the image acquisition procedure.

**Fig. 3:**
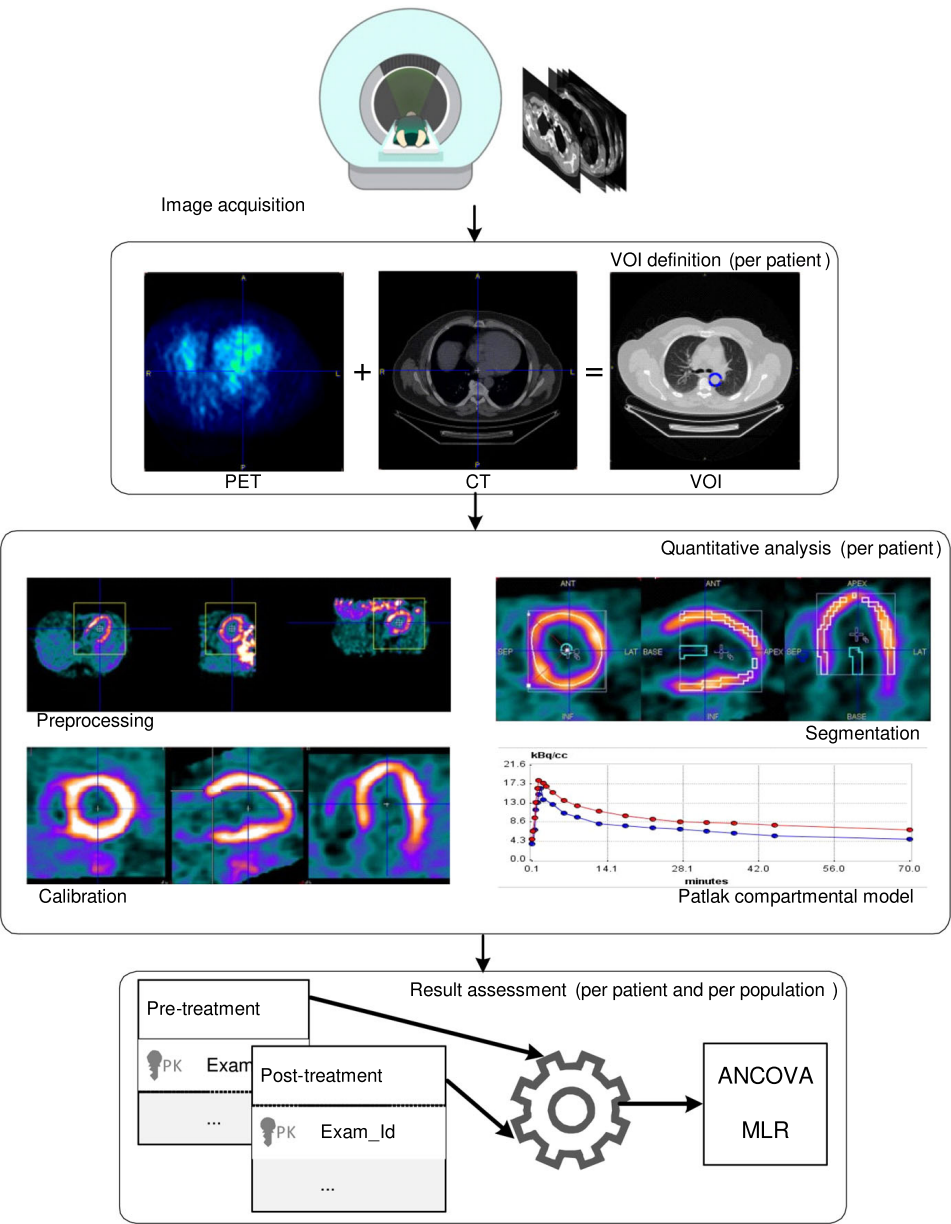
An example of using the proposed methodology for MRGlu evaluation in the myocardium and aorta districts. The VOI identification from vessels is performed via the PET and CT images. The MRGlu measurements in the myocardium and the aorta vessel during the temporal observation are obtained by using the kBecquerel/cc values (the plot in the figure). Finally, the MRGlu values are extracted and used as input to the statistical module, where the measurements from the entire set of patients are used to assess results

**Table 2:** Example of MRGlu values for aorta and myocardium, pre and post-treatment, for every patient, and the respective risk factors

Pt Id	Group	MRGlu	MRGlu	MRGlu	MRGlu	Age	LeanM	BMI		
		Aorta pre	Aorta post	Myoc pre	Myoc post					
1	1	$-$4.29	$-$0.91	2.11	22.83	57	4.98	33.01		
2	1	$-$4.75	$-$0.61	9.83	23.84	61	5.35	29.07		
3	2	$-$6.42	1.38	2.99	3.94	64	4.21	29.55		
4	2	$-$0.51	0.67	15.94	26.85	56	4.00	36.2		
W	sBP	dBP	PP	FPG	FPIns	ESR	PCR	PWV	Ao	Pt Id
116	128	90	38	116	10	5	2.75	7	4.47	1
103	110	70	40	128	7.6	7	2.18	5.2	2.6	2
100	110	60	50	156	26	38	3.23	6.8	3.5	3
118	140	80	60	112	21	9	0.86	7.3	3.4	4

The four steps of the proposed methodology (see Fig. 1) have been followed on the considered dataset. Thus, Fig. 3 represents an instance of the proposed model on one single patient. The application of the proposed model for all patients of Group1 and Group2 allows us to obtain the MRGlu values dynamic evolution. Values are then used to perform statistical analysis on the entire population (i.e., two groups of patients) to provide physicians with information regarding the effects of the drug protocol. Table 2 reports an example of obtained results where MRGlu values are associated with risk factors of Table 1 for the considered 20 patients.

**Fig. 4:**
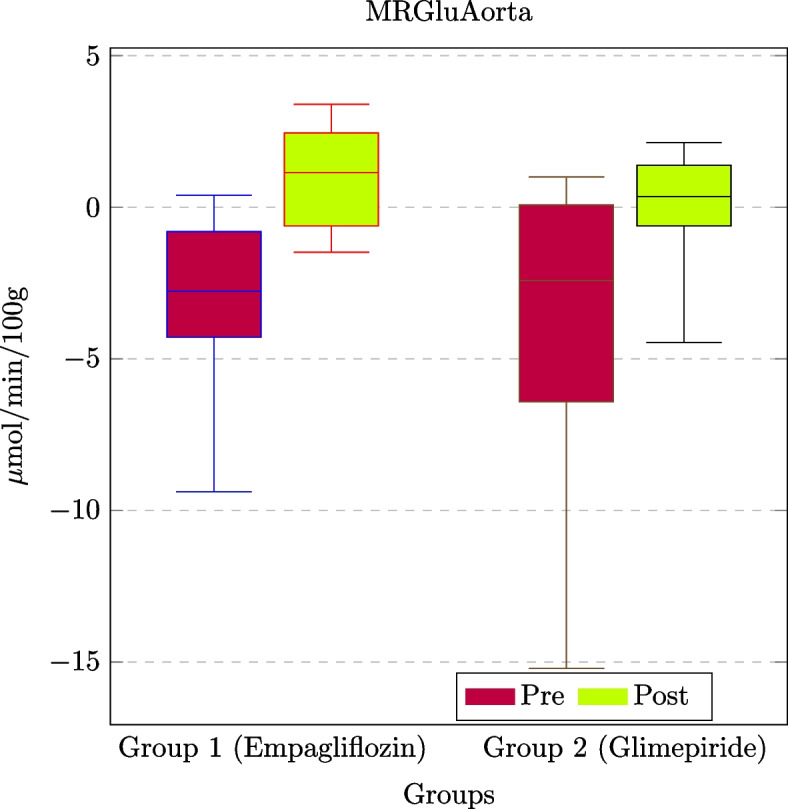
Box plot of the behavior of MRGluAorta for the administration of the two drugs over the two patient groups (empagliflozin for *Group*1 and glimepiride for *Group*2, respectively)

The application of the workflow of Fig. 3 to the entire population returns MRGlu values for the aorta and myocardium district of each patient. Experimental results are then used as input for the statistical comparison of metabolic glucose rate values (see bottom part of Fig. 1).

Figure 4 depicts the MRGluAorta values in both *Group*1 and *Group*2, in pre-treatment (e.g., baseline) and post-treatment conditions. Negative values for MRGlu are filtered out from the clinical analysis. Such values, according to the literature, result from the application of the compartmental model instance. The negative values of MRGlu derive from the Patlak model and are caused by the slope of the input function and by 18F-FDG concentration in the analyzed VOI. Thus, negative values in the model represent the concentration of the 18F-FDG in the volume of interest with respect to the input function generated by the VOI of the left ventricle [40].

**Table 3:** The statistical metrics for both groups (*Group1* and *Group2*) and for both conditions (*pre* and *post* treatment) have been extracted

Group	Group1		Group2		Group1		Group2	
	Pre	Post	Pre	Post	Pre	Post	Pre	Post
Sample size (*n*)	10	10	10	10	10	10	10	10
Minimum	$-$9.3891	$-$1.4886	$-$15.2059	$-$4.4641	$-$0.8913	0.1034	0.725	$-$0.5012
Q1	$-$4.2895	$-$0.6151	$-$6.4201	$-$0.6126	2.1087	9.0616	2.798	2.1351
Median	$-$2.7691	1.1469	$-$2.4224	0.3481	8.2105	17.0434	3.7309	6.6774
Q3	$-$0.8013	2.4497	0.0746	1.3798	21.7759	22.4931	26.8847	24.8354
Maximum	0.3901	3.3928	0.9955	2.1346	33.241	23.8391	29.358	26.8557
Mean	$-$2.9934	1.0952	$-$3.9922	$-$0.2404	11.9966	14.8512	11.5002	11.3686
Skewness	$-$1.0041	$-$0.1878	$-$1.2432	$-$1.1755	09757	$-$0.8155	0.7014	0.5151

All values are expressed in $\mu mol/min/100g$

**Fig. 5:**
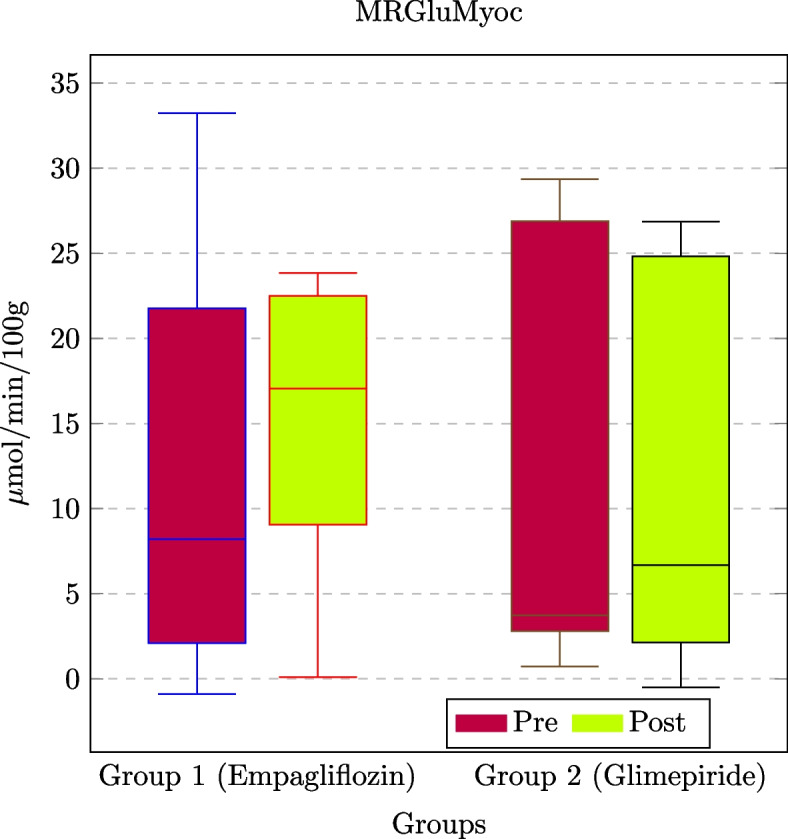
Box plot of the behavior of MRGluMyoc for the administration of the two drugs over the two patient groups (empagliflozin for *Group*1 and glimepiride for *Group*2, respectively)

The quantitative analysis from the methodology allowed us to measure the trend of the MRGlu in the aorta (MRGluAorta) w.r.t. the MRGlu in the myocardium (MRGluMyoc). Table 3 reports the measured mean values for MRGlu detected in both aorta and myocardium (where MRGlu values are expressed in $\mu mol/100 g/min$), allowing clinicians to obtain clinically related results. The implemented methodology also allows one to plot results as reported in Figs. 4 and 5, which graphically depict the MRGlu values of Table 3.

**Table 4:** ANCOVA test results for the 20 patients

Administered drug	District	Pre	Post	Delta	*p*-value
Empagliflozin	MRGluAorta	$-2.99 \pm 2.94$	$1.09 \pm 1.73$	$4.08 \pm 3.80$	$<0.0001*$
	MRGluMyoc	$11.99 \pm 11.91$	$14.85 \pm 8.32$	$2.86 \pm 13.12$	$0.0272*$
Glimepiride	MRGluAorta	$-3.99 \pm 5.16$	$-0.24 \pm 2.19$	$3.75 \pm 5.04$	$0.0019*$
	MRGluMyoc	$11.50 \pm 12.17$	$11.36 \pm 11.11$	$-0.14 \pm 8.68$	$0.0337*$

All values are expressed in $\mu mol/min/100g$. Pre- and post-columns contain pre- and post-treatment values for the entire population (expressed as mean± standard deviation); the delta column contains differences between the post and pre-values. Such indexes are used by physicians to evaluate the efficacy of the treatments. In particular, the results obtained by using the proposed methodology allowed the physicians to claim that different drug administrations modified the MRGlu values in both districts

Figure 5 compares the MRGluMyoc values in both patient groups. For example, MRGluMyoc is similar for *Group*1 and *Group*2 in both conditions (e.g., pre- and post-drug administration). The obtained plots of Figs. 4 and 5 support clinicians in studying the MRGlu values trend with respect to treatment effects. For instance, the reported example allowed them to observe that after 6 months of treatment, the MRGlu values increased in both groups.

The statistical module allowed us to validate the clinical hypothesis in terms of the correlation between predicted and observed data concerning drug administration. In particular, the application of the ANCOVA inferential test from the methodology returned the values reported in Table 4. Such values allowed clinicians to validate the study claiming that the application of empagliflozin and glimepiride drugs present significant effects on MRGlu values in the two analyzed districts [39].

Besides the statistical variables over the distribution of the data (mean, median, skewness, etc.), the methodology also provides one statistical inferential test on the entire set of studied patients. The multiple linear regression module allows one to analyze the correlation between the MRGlu values and patient risk factors (see Table 1).

**Table 5:** Results of the multiple linear regression analysis for MRGlu values both in aorta and myocardium districts to analyze their correlation with patient risk factors

District	*F*-statistic	*p*-value	*R*
MRGluAorta	21.6537	0.000006	0.9626
MRGluMyoc	12.8319	0.000102	0.9392

Table 5 reports the results of the multiple linear regression module of the proposed methodology. The *F*-statistic, *p*-value, and the multiple correlation coefficient (*R*) represent the reliability of using the independent variables (i.e., the risk factors) to estimate the dependent variable (i.e., MRGlu). In the considered set of patients, the *R* value in Table 5 is 0.9626 for MRGlu values in the aorta vessel, indicating a high capability of risk factors to predict MRGlu value. Results proved that there is a significant relationship between the independent variables (risk factors) used in the clinical experimental analysis and the dependent variable (MRGlu). Thus, the MRGlu values from districts different from the myocardium can be used to predict metabolic uptake. Even if the experiments have been performed in the heart-related district, the methodology [41] can be used in a more general-purpose way by selecting districts and using PET and CT images. Indeed, an increasing value in FDG uptake in patients with diabetes can be associated with an increased inflammatory state.

## Discussion and Conclusion

We proposed a methodology to evaluate metabolic glucose rate (MRGlu) in human districts. The methodology exploits PET and CT images with a mathematical model and a statistical module. The methodology involves the extraction of different volumes of interest (VOIs) to quantify and assess the progression of glucose metabolism in those VOIs. We applied the methodology to a real use case, choosing as VOIs the myocardium and the aorta districts. Results have been clinically validated, too.

The application of the methodology in a real scenario allowed us to achieve a twofold validation: (i) we validated the efficacy of the proposed methodology, and (ii) we validated the clinical hypothesis of a statistical correlation between MRGlu values and cardiovascular risk factors, also proving that the proposed methodology can be used to evaluate the incidence of cardiovascular risk indexes in diabetic patients. The methodology has been used in previously proposed clinical studies [39]. To assess myocardial and whole-body metabolism, the use of cardiac PET combined with the euglycemic-hyperinsulinemic clamp technique is considered the gold standard method in clinical studies. However, such a method, being based on clamping, requires standardized training, a skilled staff, and a qualified clinical center. This may result in a limitation to using the proposed methodology in a large-scale clinical context.

The major novelty of our approach consists of defining a new methodology, to measure and identify MRGlu values in biological tissues, by merging bioimages from different sources. The methodology is an improvement in terms of clinical support in studying direct drug effects on dysmetabolic patients. Results proved that the methodology can successfully evaluate the effects of treatments for patients with metabolic disorders and cardiovascular risk factors. In order to prove its effectiveness, the methodology has been tested on a cohort of 20 patients affected by type 2 diabetes.

The methodology is currently in use at the dysmetabolic clinical unit of the University of Catanzaro Medical Hospital. Indeed, the methodology allows clinicians to measure the correlation among pathology, clinical protocol, and cardiovascular risk indexes. These indexes can be used to predict changes in glucose metabolism in heart-related districts. For example, Table 5 shows how the metabolism change is strongly related to cardiovascular risk factors (e.g., for MRGluAorta, the multiple correlation coefficient *R* is 0.926 with a *p*-value of 0.000006). Finally, further details about the proposed methodology can be found at [41].

For future research activities, we plan the following:

i. To couple the methodology to a standardized protocol. By a standardized protocol, several research centers may cooperate and share data from a bigger number of patients: this could benefit in achieving other clinically relevant results on a wider cohort of patients.
ii. To use the methodology to study damages from the CoViD-19 infection. The methodology can help in comparing the images of a patient before and after the infection by CoViD-19, obviously in a retrospective way. The methodology can be used to compare PET/CT images already acquired before the infection, with PET/CT images acquired after the infection. Images before and after the infection can be used as input to the methodology to evaluate the residual effects on the metabolism of the patient as originated by the infection itself.
iii. To extend the methodology to a general-purpose framework where PET/CT merging can be used to analyze tissues’ responses to drug protocols.
iv. To perform a full kinetic analysis, using images derived from cardiac districts (e.g., aorta) as input functions to the compartment model (e.g., the Patlak model in our case).

## Data Availability

Acquired data are made available upon request to reviewers, only.

## Code Availability

The workflow mechanism is freely available at the GitHub repository [41].
